# Fasting mimicking diet during neo-adjuvant chemotherapy in breast cancer patients: a randomized controlled trial study

**DOI:** 10.3389/fnut.2024.1483707

**Published:** 2024-12-04

**Authors:** Alireza Bahrami, Shirin Haghighi, Mona Malekzadeh Moghani, Nastaran Khodakarim, Ehsan Hejazi

**Affiliations:** ^1^Department of Clinical Nutrition and Dietetics, School of Nutrition Sciences and Food Technology, Shahid Beheshti University of Medical Sciences, Tehran, Iran; ^2^Department of Oncology, Gastroenterology and Liver Disease Research Center, Shahid Beheshti University of Medical Sciences, Tehran, Iran; ^3^Department of Radiation Oncology, Shohada-e-Tajrish Hospital, Shahid Beheshti University of Medical Sciences, Tehran, Iran; ^4^Department of Hematology & Oncology, School of Medicine, Iran University of Medical Sciences, Tehran, Iran

**Keywords:** fasting mimicking diet, fasting, chemotherapy, breast cancer, toxicity

## Abstract

**Objective:**

Preclinical evidences suggests that while fasting can reduce the side effects and toxicity of chemotherapy, it can make cancer cells more susceptible to chemotherapy. This study aimed to examine the effects of fasting mimicking diet (FMD) during neo-adjuvant chemotherapy in breast cancer (BC) patients.

**Methods:**

Forty-four newly diagnosed human epidermal growth factor receptor 2-negative (HER2-negative) patients with BC were randomized equally into two groups (22 each), to receive either a fasting mimicking diet (FMD) or their regular diet for 3 days prior to and during neoadjuvant chemotherapy. This FMD was repeated every 3 weeks for 8 cycles. Efficacy, toxicity, hematologic, metabolic, and inflammatory parameters were measured and compared.

**Results:**

The occurrence of grade III vomiting and neutropenia in the control group was significantly higher than the FMD group (*P* = <0.001 and *p* = 0.04 respectively). Erythrocytes (*p* = 0.01) and neutrophils (*p* = 0.002) counts were significantly higher in FMD group compared to control group after cycle 8. There was a significant increase in median glucose and median insulin levels (*p* = 0.01 and *p* = 0.005, respectively) in the control group between baseline and after cycle 8. While, the median Insulin-like growth factor-1 (IGF1) (*p* = 0.006) and hs-CRP (*p* = 0.02) levels were significantly decreased in the FMD group. At the end of study (after cycle 8), the median glucose level was significantly higher in control group (*p* = 0.008), while the median hs-CRP level was significantly lower in FMD group (*p* = 0.01). The Miller and Payne pathological response 4/5 (90–100% tumor cell loss) and the radiologically complete or partial response, as measured by MRI or ultrasound before surgery occurred more frequently in FMD group compared to the controls (*p* = 0.01).

**Conclusion:**

Fasting mimicking diet was well tolerated during chemotherapy and reduced toxicity of chemotherapy and also, had beneficial effects of some metabolic parameters.

**Clinical Trial Registration:**

https://irct.behdasht.gov.ir/user/trial/61386/view.

## Introduction

Breast cancer (BC) is the most common cancer and the leading cause of cancer-related deaths among women in the world (1). The incidence of BC in Iran has had an increasing trend in recent years. Among Iranian women, BC is the most common cancer and in recent years the age of onset has decreased by about 10 years (from 40.0 to 30.0 years) also it is predicted that 7,000 new cases of breast cancer will be detected every year in Iran (2, 3). Although chemotherapy and radiotherapy combined with surgery are still effective treatments and improve the survival rate of cancer patients, they lead to damage to healthy tissues and significant side effects such as psychological distress, fatigue, vomiting, diarrhea and even death (4, 5). In the last decade, it has been shown that some dietary changes may lead to treatment and decrease cancer incidence (6). Previous studies show that calorie restriction reduces the incidence of cancer and mortality and delays the progression of tumors (7–9). Calorie restriction can be done through an overall reduction in dietary calories or by short-term and intermittent fasting periods (10). Recent evidences suggest that short-term fasting and fasting mimicking diets (FMDs) can make cancer cells more susceptible to respond to chemotherapy while protecting healthy cells against the harmful effects of chemotherapy (11–13). Fasting signals healthy cells to regulate their proliferating state and switch to maintenance and repair state which causes resistance to chemotherapy, whereas oncogenic mutation makes tumor cells unable to activate this protective pathway. Therefore, nutrient scarce conditions render cancer cells more susceptible to cancer therapy. This phenomenon known as differential stress resistance (DSR) (14, 15). FMD has been developed as a low-calorie, low-protein strategy to mimic the fasting state, which has been reported reduce plasma levels of insulin-like growth factor 1 (IGF-1), insulin, and glucose as mediators of cancer cell growth and progression (12, 16). Some previous studies revealed that fasting in combination with chemotherapy is safe and feasible and may decrease chemotherapy-induced toxicity and side effects (17–20). The aim of present study was to determine the impact of FMD on toxicity and metabolic change during neo-adjuvant chemotherapy in BC patients.

## Method

### Patients

**Figure 1:**
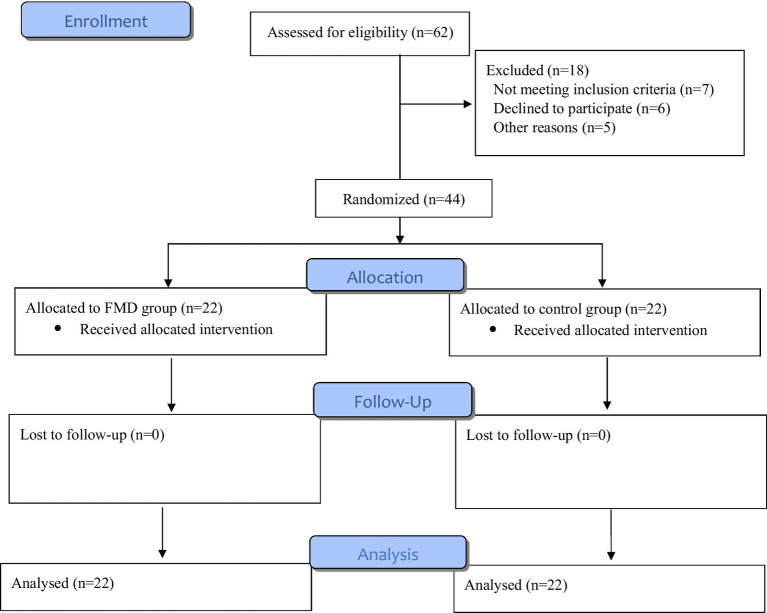
Flow diagram of the trial process.

In this randomized controlled study, we recruited 44 recently diagnosed HER-2 negative BC patients between February 2022 and January 2024 from Taleghani Hospital, Tehran, Iran (Figure 1). Our inclusion criteria were: age ≥ 18 years; WHO performance status 0–1; expectancy of life >3 months; adequate bone marrow function (i.e., white blood counts >3.0 × 109/L, absolute neutrophil count ≥1.5 × 109/L and platelet count ≥100 × 109/l); adequate liver function, adequate renal function, normal cardiac function, and no allergy to FMD; absence of diabetes mellitus; absence of pregnancy or current lactation; and signed informed consent form. This study was approved by the Ethics Committee of the Faculty of Nutrition and Food Technology, Shahid Beheshti University of Medical Sciences, Tehran, Iran (IR.SBMU.NNFTRI.REC.1400.062). We also registered the RCT at www.irct.ir (IRCT20171227038099N1). Before enrollment in the RCT, all the patients had signed informed consent form.

### Intervention

Patients were randomized in a 1:1 ratio to receive the FMD regime with 2-grams Omega3 or regular diet for 3 days before to and on the day of each cycle of chemotherapy. One of the reasons for lack of adherence to the experimental FMD diet was hunger and lethargy of patients due to low calorie content which providing ~1,200 kcal on day 1 and ~200 kcal on days 2–4 (12). Accordingly, a modified-FMD consisting of soups (Elite^®^ soups; Vermicelli, mushroom, tomato), Mani^®^ bar, liquids and tea (Supplementary Tables S1, S2) was used in the present study. Calorie content reduced from day 1 (~1,100 kcal), to days 2–4 (~500 kcal). Moreover, the percent of carbohydrates/proteins/fats from total energy was approximately 53, 13 and 34 on the first day, and 53, 9 and 38 on the other subsequent 3 days. Patients were allowed to consume their FMD diet at any time of the day. After the completion of the first course, another package containing soup and supplements for the second course was delivered to the participants. Participants’ compliance was assessed by asking them to record all foods consumed during the 4-day FMD and to return empty packets of soup and supplements at the end of each cycle.

### Drugs

On the first day of each 3-weekly cycle (8 in total), patients received neo-adjuvant chemotherapy, consisting of 4 cycles of Adriamycin and cyclophosphamide at a dose of 60, and 600 mg m^−2^ i.v., respectively, followed by 4 cycles of Paclitaxel (175 mg m^−2^). The anti-emetic agents granisetron (1 mg i.v.) or ondansetron (8 mg i.v.) were administered prior to chemotherapy. Dexamethasone (8 mg in the first 4 cycles and 16 mg for the second 4 cycles i.v.) was administered shortly before chemotherapy to prevent hypersensitivity reactions and fluid retention prior to chemotherapy infusions.

### Randomization

Patients were randomized through block randomization stratified by estrogen receptor status (positive versus negative) and tumor stage (II versus III).

### Blood sampling

Venous blood samples were collected in fasting state 2 days before starting chemotherapy (baseline), and immediately at the end of the eight cycle of chemotherapy protocol. The effects of FMD were assessed by evaluating (1) Metabolic parameters (insulin, glucose, and IGF-1); (2) Hematologic parameters (erythrocyte-, platelet and leukocyte count); (3) Inflammatory marker [Highly sensitive C-reactive protein (hs-CRP)]. For measurement of metabolic parameters, blood samples were drawn in serum-separating tubes, allowed 20 min for coagulation, then centrifuged at 4,000 r.p.m. for 10 min. Serum was separated and stored at −80°C until analysis. Human insulin was measured by an enzyme-linked immunosorbent assay (ELISA) kit [Perfect Ease Biotech (Beijing) Co.]. Human insulin-like growth factors 1 (IGF-1) and was measured by ELISA kits (Sun Red Company, China, 201–11-0710). Hs-CRP was also assessed by ELISA kit (ZillBio, Germany, ZX-55114-96).

### Toxicity and efficacy

During each cycle, participants were asked to report the experienced side effects, graded as mild, moderate or severe. Self-reported side effects, side effects documented by the physician and hematological toxicity were graded according to the Common Terminology Criteria for Adverse Events version 5 (CTCAE v.5) (21). Two methods were used to evaluate the efficacy of chemotherapy; the pathological response reported by a blinded pathologist based on Miller and Payne’s method and clinical response was measured by MRI or ultrasound of the breast halfway and at the end of therapy, according to RECIST1.1 (22, 23).

### Assessment of paraclinical variables

All participants were measured for anthropometric indices including weight, height, and waist at baseline and at the end of the study. Measurements were taken on standard scales, without shoes and with minimal clothing on the subjects. Body mass index (BMI) computed as weight (in kilograms)/square of height (in meters). A short form International Physical Activity Questionnaire (IPAQ) was used to assess physical activity at baseline and the end of intervention (24).

### Statistical analysis

Statistical tests were performed using SPSS software (v.21). Significance level was set at *p* < 0.05, and all *p-*values were based on two-sided tests. The Kolmogorov–Smirnoff test was used to evaluate whether or not the distribution of the variables was normal. The mean values of two groups were compared using the student’s t-test and the means for normal distribution variables. Also, non-parametric statistics, including the Mann–Whitney *U* test or Kruskal–Wallis test were used for variables without normal distribution. Moreover, the chi-square test was used for comparing categorical variables. The Wilcoxon signed-ranks test for abnormally distributed quantitative data and the paired t-test for regularly distributed quantitative values of the variables were used to compare the two periods.

## Results

Forty-four patients aged between 35 and 68 years were randomly divided into two groups (22 patients in each group). Mean age of participants was 49.36 ± 8.19 and 50.59 ± 8.59 years for FMD and control groups, respectively. Baseline characteristics of participants were provided in Table 1. Regarding demographic and biological characteristics no significant difference was observed between the two groups. All patients finished 8 cycle of chemotherapy protocol.

**Table 1:** General characteristics of the participants.

Variables	FMD group (*n* = 22)	Control group (*n* = 22)	*P*
Age (Mean ± SD)	49.36 ± 8.19	50.59 ± 8.59	0.63
BMI (Mean ± SD)	21.38 ± 1.82	21.51 ± 2.19	0.82
Educational level *n* (%)			0.31
Illiterate	2 (9.1)	0 (0)	
Low education	16 (72.7)	16 (72.7)	
High education	4 (18.2)	6 (27.3)	
Smoking (yes) *n* (%)	7 (31.8)	5 (22.7)	0.49
Family history of cancer in first degree (yes) *n* (%)	8 (36.4)	6 (27.3)	0.51
Menopausal status *n* (%)			0.75
Pre	7 (31.8)	8 (36.4)	
Post	15 (68.2)	14 (63.6)	
WHO Status *n* (%)			0.72
0	17 (77.3)	16 (72.7)	
1	5 (22.7)	6 (27.3)	
ER status *n* (%)			0.36
Positive	9 (40.9)	12 (54.5)	
Negative	13 (59.1)	10 (45.5)	
PR status *n* (%)			0.24
Positive	11 (50)	13 (59.1)	
Negative	11 (50)	9 (40.9)	
Tumor type *n* (%)			0.13
Ductal	9 (40.9)	14 (63.6)	
Lobular	13 (59.1)	8 (36.4)	
Tumor grade *n* (%)			0.51
II	14 (63.6)	16 (72.7)	
III	8 (36.4)	6 (27.3)	
Tumor stage *n* (%)			0.53
II	7 (31.8)	9 (40.9)	
III	15 (68.2)	13 (59.1)	
Physical activity (Mean ± SD) (met/h/day)	36.82 ± 2.4	37.55 ± 2.13	0.29

Independent sample *t*-test was used for continuous variables and Chi-square was used for categorical variables. BMI, Body Mass Index; ER, Estrogen Receptor; PR, Progesterone Receptor; HER2, Human Epidermal Receptor 2; WHO, World Health Organization; FMD, fasting mimicking diet; MET, metabolic equivalent.

### Toxicity

Grades I–III had the highest observed toxicity among patients. The details of the percentage of occurrence and grading of each side effect of toxicity are shown in Table 2. No patient experienced grade IV or V toxicity during chemotherapy in either group. The occurrence of grade III vomiting and neutropenia in the control group was significantly higher than the FMD group (*P* = <0.001 and *p* = 0.04 respectively). No significant difference was observed between the two patient groups in the occurrence of diarrhea, nausea, constipation, thrombocytosis, or mouth sores.

**Table 2:** Grading toxicity during 8 cycles of chemotherapy between two groups.

Toxicity	FMD group (*n* = 22)			Control group (*n* = 22)			*P* *
	Grade I	Grade II	Grade III	Grade I	Grade II	Grade III	
Diarrhea *N* (%)	10 (45.5)	9 (40.9)	3 (13.6)	10 (45.5)	7 (31.8)	5 (22.7)	0.68
Vomiting *N* (%)	4 (18.2)	15 (68.1)	3 (13.6)	3 (13.6)	7 (31.8)	12 (54.5)	<0.001
Nausea *N* (%)	10 (45.5)	8 (36.4)	4 (18.2)	3 (13.6)	10 (45.5)	9 (40.9)	0.16
Constipation *N* (%)	10 (45.5)	11 (50)	1 (4.5)	10 (45.5)	9 (40.9)	3 (13.6)	0.91
Neutropenia *N* (%)	6 (27.2)	11 (50)	5 (22.7)	5 (22.7)	7 (31.8)	10 (45.5)	0.048
Thrombocytosis *N* (%)	15 (68.2)	6 (27.3)	1 (4.5)	15 (68.2)	6 (27.3)	1 (4.5)	1.00
Mouth sores *N* (%)	4 (18.2)	11 (50)	7 (31.8)	1 (4.5)	11 (50)	10 (45.5)	0.31

* Fisher Exact test was used to compare between FMD group and control group.

### Hematologic parameters

According to Table 3, erythrocytes, hemoglobin, white blood cells, and neutrophils were decreased, while platelets were increased after 8 cycle compared to baseline in both groups. Erythrocytes and neutrophils counts were significantly higher in FMD group compared to control group after cycle 8 (*p* = 0.01 and *p* = 0.002 respectively).

**Table 3:** Comparison of hematologic parameters within and between two groups after cycle 8.

Parameters	FMD group (*n* = 22)			Control group (*n* = 22)			*P* ^¶^
	Baseline	After cycle 8	P*	Baseline	After cycle 8	P*	
RBC (×10^6^/μl)	4.22 ± 0.53	4.06 ± 0.37	0.007	4.12 ± 0.48	3.79 ± 0.34	0.01	0.01
HG (g/dl)	12.39 ± 0.79	12.15 ± 0.72	0.01	12.45 ± 0.79	12.21 ± 0.74	0.006	0.83
WBC (×10^3^/μl)	4.86 ± 0.73	4.55 ± 0.92	0.006	4.91 ± 0.77	4.56 ± 0.93	0.004	0.94
PLT (×10^3^/μl)	302.72 ± 45	316.77 ± 54.1	0.03	305.5 ± 44.5	322.04 ± 53.1	0.01	0.74
NEUT (×10^3^/mm^3^)	4.61 ± 0.65	3.98 ± 0.48	0.001	4.57 ± 0.73	3.58 ± 0.31	0.001	0.002

Data presented as Mean (SD). *Within group (comparing between baseline and after cycle 8), Paired *t*-test was used. ^¶^Between group (comparing between FMD group and control group), independent *t*-test was used. RBC, red blood cells; HG, hemoglobin; PLT, platelets; WBC, white blood cells; NEUT, neutrophils.

### Metabolic and anthropometric parameters

Between baseline and after cycle 8, median glucose and insulin levels were significantly increased in control group (*p* = 0.01 and *p* = 0.005 respectively), while there was no significant difference in glucose and insulin levels in FMD group. On the other hand, the median levels of IGF-1 and hs-CRP was significantly decreased in FMD group between the two-time points (*p* = 0.006 and *p* = 0.02 respectively) (Table 4). At the end of study (after cycle 8), the median glucose level was significantly higher in control group (*p* = 0.008) compared to the FMD group, while the median hs-CRP level was significantly lower in FMD group (*p* = 0.01) than those in the control group. There was no significant difference in IGF1 and insulin levels, weight and BMI between the two groups (data not shown).

**Table 4:** Comparison of metabolic and anthropometric parameters within and between two groups after cycle 8.

Parameters	FMD group (*n* = 22)			Control group (*n* = 22)			*P* ^¶^
	Baseline	After cycle 8	P*	Baseline	After cycle 8	P*	
Glucose (mg/dl)	97 (22)	97.5 (14)	0.38	97.5 (24.5)	106 (12.38)	0.01	0.008
Insulin (μU/ml)	14 (12.6)	12.3 (6.91)	0.25	12.6 (7.93)	12.85 (11.25)	0.005	0.51
IGF-1 (ng/ml)	181 (30.5)	172 (27.5)	0.006	181 (30.5)	181 (22.75)	0.09	0.64
hs-CRP (mg/l)	4.61 (2.16)	3.49 (2.07)	0.02	5.7 (2.81)	5.15 (3.21)	0.11	0.01
Weight (kg)	60.5 (6)	59.5 (7)	0.77	59 (5)	59.8 (5.73)	0.85	0.88
BMI (kg/m^2^)	21.2 (2.68)	21 (2.74)	0.73	21.1 (2.62)	21.2 (2.59)	0.81	0.79

Data presented as Median (IQR). *Within group (comparing between baseline and after cycle 8), Paired *t*-test was used. ^¶^Between group (comparing between FMD group and control group), independent *t*-test was used. IGF1, insulin-like growth factor-1. Hs-CRP, highly sensitive C-reactive protein. BMI, Body mass index.

### Efficacy

The efficacy of chemotherapy based on radiological and pathological response is shown in Table 5. According to this table, the Miller and Payne pathological response 4/5 (90–100% tumor cell loss) and the radiologically complete or partial response, as measured by MRI or ultrasound before surgery occurred more frequently in FMD group compared to the controls (*p* = 0.01).

**Table 5:** Efficacy of chemotherapy based on tumor response data.

Efficacy	FMD group		Control group		*P* *
Pathological response according to Miller and Payne *N* (%)	Score 1/2/3	Score 4/5	Score 1/2/3	Score 4/5	0.03
	6 (27.3)	16 (72.7)	13 (59)	9 (41)	
Clinical response according to MRI or ultrasound *N* (%)	CR/PR	SD/PD	CR/PR	SD/PD	0.03
	15 (68.1)	7 (31.9)	8 (36.3)	14 (63.7)	

* Fisher Exact test was used to compare between FMD group and control group. The pathological response was given for Miller and Payne pathological response score 4/5 (90–100% tumor cell loss) vs. 1/2/3 (less than 90% tumor cell loss). The radiological response was scored according to RECIST 1.1 and given for complete response (CR) and partial response (PR) vs. stable disease (SD) and progression disease (PD).

## Discussion

This randomized controlled study showed that FMD was well-tolerated, safe and had beneficial effects on chemotherapy-related toxicity, hematologic and metabolic parameters. All subjects completed the treatment schedule and no one dropped out. Side effects related to chemotherapy in the present study were similar to those reported in previous studies (25–27). Our findings showed that the occurrence of grade III vomiting and neutropenia in the control group was significantly higher than in the FMD group. The reduction of vomiting in the FMD group could be due to the induction of the DSR mechanism in fasting condition, in which the cells of the gastrointestinal tract (GIT) are also protected from destruction by chemotherapy drugs (28, 29). Consistently with our findings, the results of a study by Omar et al. showed a significant decrease in nausea, vomiting, diarrhea and mouth sores in the fasting group (30). In another case series study, Safdie et al. (29) observed that during the fasting cycles, none of the gastrointestinal side effects were reported in the patients undergoing chemotherapy. Our results regarding hematologic parameters showed that erythrocytes and neutrophils count significantly higher in the FMD group compared to control group after 8th cycle. In agreement with our results, de Groot et al. (27) observed a higher number of erythrocytes after chemotherapy in the fasting group compared to the control group, although the number of neutrophils was not significantly different between the two groups. The increase in the number of erythrocytes and neutrophils in the FMD group may be due to the decrease in the breakdown of circulating cells and severe bone marrow suppression in fasting conditions. This could strengthen the hypothesis that fasting diet such as FMD may protect healthy cells against chemotherapy-induced hematologic toxicity (12, 27). Glucose and insulin levels in the control group increased significantly between the baseline and after cycle 8, while there was no significant difference in the mean glucose and insulin levels in the FMD group. In addition, there was a significant increase in plasma glucose levels in the control group compared to the FMD group after 8th cycle. Despite the dexamethasone injection in each chemotherapy cycle, glucose levels were lower in the intervention group compared to the control group, indicating a possible effect of the FMD diet, which is high in complex carbohydrates and low in sugar and calories. Findings obtained by de Groot et al. (12) showed that compared to the beginning of the study, the plasma glucose trend in the FMD group was significantly lower than in the control group. However, in this study, dexamethasone was omitted in the FMD group (12). In agreement with our results, Omar et al. (30) reported that serum glucose and insulin levels were significantly higher in control group compared to fasting group after 4th cycle of chemotherapy. Also, a clinical trial on 101 patients with different tumor types and treated with concomitant antitumor therapies indicated that a cyclic, 5 days FMD resulted in a persistent reduction of blood glucose and growth factor concentration (31). However, contrary to our findings another study by de Groot et al. (27) indicated that plasma glucose levels significantly increased in the fasting group after 6th cycle of chemotherapy which was explained by the use of dexamethasone. In the present study, the serum level of IGF-1 in the FMD group decreased significantly between the baseline and after cycle 8. Previous clinical data shows that reduced serum glucose and insulin levels are associated with a better prognosis of breast cancer in non-diabetic patients (27, 32). Recent evidence suggests that the combination of FMD and hormone therapy in hormone receptor-positive breast cancer induces metabolic changes such as decreased insulin and IGF-1 levels for a long time, which can lead to long-term anticancer activity (33). On the other hand, reduction in IGF-1 levels could downregulate cell growth and proliferation by affects on other factors such as mammalian target of rapamycin (mTOR), Akt and Ras (34, 35). These mechanisms can promote DSR, protect healthy cells against chemotherapy, and strengthen the effect of chemotherapy drugs on cancer cells (14, 15).

Our results showed that the median level of hs-CRP decreased between the baseline and the 8th cycle of chemotherapy. Also, at the end of the study (after cycle 8), the mean level of hs-CRP in the FMD group was significantly lower than the control group. Better glycemic control, and the role of omega 3 (36) may have improved inflammatory conditions in the FMD group. CRP is a marker of inflammatory response and the reduction of CRP in the FMD group can reduce chronic inflammation, oxidative stress, and ultimately reduce the progression of cancer cells (37, 38).

Our study has some limitation. First, the small sample size of our study which may have limited the power of study. Second, the possibility of incomplete adherence to FMD schedule. However, participants’ compliance was assessed by asking them to record all foods consumed during the 4-day FMD and to return empty packets of soup and supplements at the end of each cycle. Also, the injection of dexamethasone may counteract the beneficial effects of FMD in the present study, because dexamethasone causes hyperglycemia, compensatory hyperinsulinemia, and eventually insulin resistance. Moreover, Due to the short observation period, it was not possible to evaluate the effect of FMD on prognosis.

## Conclusion

The present study demonstrated that a cyclic FMD program is safe and feasible in BC patients undergoing neo-adjuvant chemotherapy. Also, the findings of the current trial provide evidence that FMD may increase the efficacy of chemotherapy and reduce some chemotherapy-induced gastrointestinal and hematologic toxicity and have a beneficial effect on some metabolic parameters.

## Data Availability

The raw data supporting the conclusions of this article will be made available by the authors, without undue reservation.
