# Supplementary material for: Fasting mimicking diet during neo-adjuvant chemotherapy in breast cancer patients: a randomized controlled trial study
Source: Front Nutr. 2024 Dec 4;11:1483707. doi: 10.3389/fnut.2024.1483707 (PMC11656309; doi:10.3389/fnut.2024.1483707)
Supplement: Supplementary file 1 [file Table_1.DOCX]

| Diet schedule | Day 1 | Day 2 | Day 3 | Day 4 |
| --- | --- | --- | --- | --- |
| Breakfast | Soup 1 | Tea | Tea | Tea |
| Morning Snack | Tea + ½ Bar | - | - | - |
| Launch | Soup 2 + Multivitamin-mineral + 1 gr Omega3 | Soup 1 + 1 gr Omega3 | Soup 1 + 1 gr Omega3 | Soup 1 |
| Afternoon Snack | Tea + ½ Bar | Tea | Tea | Tea |
| Dinner | Soup 3 + 1 gr Omega3 | Soup 2 + 1 gr Omega3 | Soup 2 + 1 gr Omega3 | Soup 2 |

Supplementary **Table 1**. Fasting mimicking diet schedule.

Supplementary **Table 2**. Nutritional content of soups and bar.

| Nutritional Fact | Soup 1 | Soup 2 | Soup 3 | Bar |
| --- | --- | --- | --- | --- |
| Energy | 286.7 | 221.46 | 241.57 | 314.44 |
| Carbohydrate | 33.7 | 32.74 | 36.9 | 32.84 |
| Protein | 6.45 | 5.36 | 6.37 | 18.41 |
| Fat | 14.02 | 7.68 | 7.61 | 12.16 |
